# The Advancement Stage of Gastric Cancer and the Levels of CEA and Ca19-9 in Serum and Peritoneal Lavage

**DOI:** 10.3390/biomedicines12112584

**Published:** 2024-11-12

**Authors:** Michał Bąk, Magdalena Wojciech, Adrianna Pielech, Sylwia Holka, Marek Zawadzki, Dawid Murawa

**Affiliations:** 1 General and Oncological Surgery, The Karol Marcinkowski University Hospital in Zielona Góra, 65-046 Zielona Góra, Poland; 2 Department of Surgery and Oncology, University of Zielona Góra, 65-417 Zielona Góra, Poland; 3 Institute of Mathematics, University of Zielona Góra, 65-417 Zielona Góra, Poland; 4 Department of Pathomorphology, The Karol Marcinkowski University Hospital in Zielona Góra, 65-046 Zielona Góra, Poland; 5 Faculty of Medicine, Wroclaw University of Science and Technology, 50-370 Wrocław, Poland

**Keywords:** gastric cancer, tumor markers, carcinoembryonic antigen (CEA), Ca19-9, peritoneal washings, cytology, peritoneal carcinomatosis, cancer detection

## Abstract

Background: This study aimed to analyze the relationship between the levels of tumor markers—specifically, carcinoembryonic antigen (CEA) and Ca19-9 antigen—determined in both serum (sCEA and sCa19-9) and intraoperative peritoneal washings (pCEA and pCa19-9) and the advanced stage of gastric cancer (including the occurrence of cancer cells in cytology from abdominal fluid). Methods: This study included 47 patients with histopathologically confirmed gastric cancer or gastroesophageal junction cancer who underwent surgical treatment. The material for the cytological examination and assessment of CEA and Ca19-9 concentrations in peritoneal fluid was collected intraoperatively. Later, blood was drawn to assess the CEA and Ca19-9 concentrations in blood serum. Results: There was a statistical correlation between a positive cytology result or the presence of peritoneal carcinomatosis and a positive result for the tumor markers obtained from abdominal washings. This correlation was not observed with marker levels obtained from blood serum. The pCEA marker was highly sensitive (93.3%) and specific (93.8%) for detecting cancer cells. The pCa19-9 marker was less effective in detecting cancer but matched pCEA in identifying the absence of cancer. No differences were observed in sCEA and sCA19-9 levels between patients who underwent neoadjuvant chemotherapy and those who did not receive this treatment. However, statistical analysis showed that this relationship did not apply to pCEA and pCa19-9 levels. Conclusions: Intraoperative determinations of tumor marker levels in peritoneal washings may be a predictive factor for a poor prognosis in patients with gastric cancer.

## 1. Introduction

Gastric cancer is the fifth most common cancer in the world. According to WCRF International, more than 1 million new cases of this cancer were diagnosed in 2020. The incidence rate significantly varies depending on the region. The highest incidence rates are recorded in East Asia, especially in countries such as Japan, Mongolia, South Korea, and China (Homepage—IARC, WCRF International (Supplementary Materials)).

The mortality rate for gastric cancer in 2020 was approximately 770,000. This number is projected to rise to approximately 1.3 million per year by 2040 (Homepage—IARC).

It is well known that dissemination into the peritoneal cavity is the most common form of recurrence for gastric adenocarcinoma, even after radical curative surgery [1,2,3,4].

Despite the availability of many diagnostic methods, such as computed tomography, magnetic resonance imaging, and positron emission tomography, none have shown a high predictive value, and a precise diagnosis of peritoneal dissemination remains a major challenge [1,2,3].

Diagnostic laparoscopy is also employed to identify peritoneal dissemination that is not visible in other radiological examinations. Cytological evaluation is also performed as part of laparoscopic staging, which enables the detection of free cancer cells before the appearance of macroscopic changes in the peritoneum, which can potentially change the treatment strategy. The detection of cancer cells via cytological examination is an accepted diagnostic method for intraperitoneal dissemination, even in the absence of peritoneal or distant metastases [5].

Although cytological examination of peritoneal lavage fluid is considered the gold standard for the detection of free tumor cells in the peritoneal cavity, its sensitivity ranges from 21% to 35%, depending on the stage of the tumors (T) and lymph nodes (N) in gastric cancer [6,7,8]. Recently, molecular approaches employing reverse transcriptase and polymerase chain reaction (RT-PCR) techniques have made it possible to increase the sensitivity of the detection of micrometastases in the peritoneal cavity [6,7,9]. However, the relatively high false-positive results, high cost of the procedure, lack of appropriate technology in all centers, and lack of immediate results during surgery are the main limitations preventing the widespread use of such techniques [6,7,9].

Unfortunately, even diagnostic laparoscopy does not ensure 100% sensitivity [10,11,12]. Leake et al. [12] evaluated the role of laparoscopy in the visual detection of peritoneal metastases in a systematic review. The accuracy, sensitivity, and specificity were 93.4–100%, 73.7–100%, and 83–100%, respectively.

Many attempts have been made over the years to investigate the relationship between tumor markers and the anatomopathological stage of gastric cancer to predict the disease course [1,13,14]. These markers are an important monitoring tool due to their easy availability and the relatively low cost of measuring their levels in serum, and they increase the observation efficiency [15]. Serum levels of carcinoembryonic antigen (CEA) and carbohydrate antigen (Ca 19-9) are associated with active gastrointestinal cancers, including gastric cancer.

It is widely accepted that measuring tumor markers before surgery in the serum and peritoneal cavity fluid of patients with gastric cancer can improve the accuracy of predicting peritoneal metastases [2,3,16].

Yamamoto’s study [2,17] confirmed that the concentration of CEA in the peritoneal fluid is a solid indicator of the early stage of peritoneal metastasis. In addition to CEA levels in the peritoneal fluid, serum CEA levels are associated with hematogenic and lymphatic metastases. However, as independent risk factors, they demonstrate low sensitivity and high false-positive values.

This study aimed to analyze the relationship between the levels of two tumor markers—carcinoembryonic antigen (CEA) and Ca19-9 antigen—measured in serum (sCEA and sCa19-9) and intraoperative peritoneal lavage fluid (pCEA and pCa19-9) and the stage of gastric cancer (including the presence of cancer cells in cytology from abdominal cavity fluid).

## 2. Materials and Methods

Forty-seven patients with histopathologically confirmed gastric or gastroesophageal junction cancer, surgically treated at the Department of General and Oncological Surgery of the University Hospital in Zielona Góra, were enrolled in this prospective study from May 2021 to May 2024.

Material for the cytological examination and measurement of CEA and Ca19-9 concentrations in the peritoneal cavity fluid was collected immediately after opening the abdominal wall. An amount of 200 mL of 0.9% NaCl was administered into the abdominal cavity at 37 °C in the area of the primary tumor. The fluid was left in the abdominal cavity for 3 min, during which the operator gently manipulated the abdominal contents. Then, 100 mL samples of the washings were collected into sterile containers and subjected to cytological examination, and 5 mL samples were collected to measure the concentrations of the CEA and Ca19-9 markers. The entire procedure, including the infusion, distribution, and fluid collection of the samples, takes about 5 min. In the case of the presence of free fluid in the peritoneal cavity, the procedure was analogous to that without free fluid but without rinsing the peritoneal cavity. The material for cytological analysis was examined urgently (the material was delivered within 15 min to the Department of Pathomorphology for examination), and part of the fluid for measuring the tumor markers was tested on an ad hoc basis. Each patient also had blood drawn to measure the concentrations of CEA and Ca19-9 in the blood serum.

The samples collected for cytological examination were centrifuged for 3 min at 1000 rpm. The remaining precipitate was smeared onto two glass slides. Each specimen was secured with Cytofix fixative and stained using the H-E method (hematoxylin and eosin) (DAKO). H-E staining is used for routine examinations. If suspicious findings, such as atypical cells, are detected, additional immunohistochemical testing is ordered. Cytological evaluation was conducted by experienced cytologists. The results of the cytological examination were considered positive when live cancer cells were present.

CEA and Ca19-9 levels were measured using a chemiluminescent enzyme-linked immunosorbent assay (Roche). Serum tumor marker levels were also measured using this method before surgery.

### Statistical Analysis

Statistical analysis was performed using the statistical software package R (R version 4.3.2) [18], which is distributed under an open-source license. Continuous variables were reported as means ± standard deviations and medians ± lower and upper quartiles. Categorical variables were expressed as frequencies and percentages. The distribution of quantitative variables was tested with the Shapiro–Wilk test. When the data were distributed normally, the equality of variance of the variables in the comparable groups was tested using the Bartlett test. The statistical significance of intergroup differences was compared using the Student’s *t*-test, Welch’s test, or the non-parametric Wilcoxon rank–sum test for continuous variables. The χ^2^ test or Fisher’s exact test was used to compare categorical variables. Fisher’s exact test of independence was used when the assumptions of the chi-square test were not held. The Wilcoxon rank–sum test with continuity correction was used when the assumptions of a normal distribution were not held. Additionally, Spearman’s rank correlation was used to assess the relationships between markers. The level of statistical significance was defined as 0.05 for all the statistical tests.

Patients were divided into different groups according to positive or negative cytology results as well as the presence or absence of visible intraperitoneal dissemination. Patient demographics and tumor characteristics, including the differentiation grade, T and N stages, and vascular, lymphatic, and neuronal invasion were analyzed. The results of morphology and kidney function (based on creatinine and GFR levels) were also included in the analysis. Patients were classified based on the sCEA and sCA19-9 levels into groups with low (≤5 ng/mL and ≤37 µ/mL) or high (>5 ng/mL and >37 µ/mL) values of these markers.

The analysis of the receiver operating characteristic (ROC) curve and the area under the curve (AUC) was used to determine the optimal cut-off values for the levels of the peritoneal tumor markers (pCEA and pCa19-9) to predict the development of tumor dissemination into the peritoneum. The definition of tumor dissemination into the peritoneum included a positive cytology result during the surgery or visible intraperitoneal dissemination. Patients were divided into groups with low or high levels of peritoneal tumor markers according to the cut-off values to assess their impact on the development of tumor dissemination into the peritoneum.

This study was approved by the Bioethics Committee of the District Medical Chamber in Zielona Góra under resolution number 10/146/2021 and followed the principles of the Declaration of Helsinki. All patients provided written informed consent to participate in this study.

## 3. Results

This study involved 47 patients with a mean age of 64 years. Of the total, 27 patients (57.4%) were male, 20 (42.6%) were female, 30 (63.8%) received neoadjuvant chemotherapy, and 1 (2.1%) received preoperative radiotherapy. Positive cytology results were reported in 11 patients (23.4%). Elevated levels of the serum tumor markers were observed in 15 (31.9%) patients for CEA and 12 (25.5%) patients for Ca19-9. Meanwhile, elevated levels of the tumor markers in the abdominal washing fluid were found in 16 (34.0%) patients for CEA and 12 (25.5%) patients for Ca19-9. The most common histological type of tumor was tubular adenocarcinoma, observed in 27 patients (57.4%), and the most common differentiation grade was G3, observed in 15 (55.6%) patients. The patient demographics, serum and peritoneal levels of tumor markers, TNM stages, other tumor characteristics, and blood counts are presented in Table 1 and Table 2.

### 3.1. Cytology—Factors Affecting a Positive Result

A positive cytology result was obtained in 11 patients (23.4%). There was no statistically significant correlation between parameters such as gender, previous neoadjuvant therapy, serum CEA and C19-9 marker levels, morphology, or kidney function (based on creatinine or GFR levels) with positive peritoneal cytology (*p* > 0.05; Table 2). Positive results were not affected by the histological type of the tumor (*p* = 0.088).

**Table 2:** Statistical relationship between the variables and cytology. The value of “1” for cytology indicates a positive cytology result, while “0” indicates a negative cytology result.

Variables, n (%)	Total	Cytology		*p*-Value
		1	0	
Number of subjects	47(100%)	11 (23.0)	36 (77.0)	
Sex				1.000
Males	27 (57.4)	6 (54.5)	21 (58.3)	
Females	20 (42.6)	5 (45.5)	15 (41.7)	
CEA in serum				0.136
Norm	32 (68.1)	5 (45.5)	27 (75.0)	
Beyond the norm	15 (31.9)	6 (54.5)	9 (25.0)	
CEA in abdominal cavity				<0.001
Norm	31 (66.0)	1 (9.1)	30 (83.3)	
Beyond the norm	16 (34.0)	10 (90.9)	6 (16.7)	
Ca19-9 in serum				0.118
Norm	35 (74.5)	6 (54.5)	29 (80.6)	
Beyond the norm	12 (25.5)	5 (45.5)	7 (19.4)	
Ca19-9 in abdominal cavity				<0.001
Norm	35 (74.5)	2 (18.2)	33 (91.7)	
Beyond the norm	12 (25.5)	9 (81.8)	3 (8.3)	
Neoadjuvant chemotherapy				0.171
1	30 (63.8)	5 (45.5)	25 (69.4)	
0	17 (36.2)	6 (54.5)	11 (30.6)	
T				0.005
0	1 (2.1)	0 (0.0)	1 (2.8)	
1	8 (17.0)	1 (9.1)	7 (19.4)	
2	3 (6.4)	0 (0.0)	3 (8.3)	
3	19 (40.4)	1 (9.1)	18 (50.0)	
4	16 (34.0)	9 (81.8)	7 (19.4)	
N				0.044
0	18 (38.3)	2 (18.2)	16 (44.4)	
1	2 (4.3)	0 (0.0)	2 (5.6)	
2	10 (21.3)	1 (9.1)	9 (25.0)	
3	17 (36.2)	8 (72.7)	9 (25.0)	
M				<0.001
0	26 (55.3)	0 (0.0)	26 (72.2)	
1	21 (44.7)	11 (100.0)	10 (27.8)	
L				0.032
0	24 (51.1)	2 (18.2)	22 (61.1)	
1	23 (48.9)	9 (81.8)	14 (38.9)	
V				0.009
0	30 (63.8)	3 (27.3)	27 (75.0)	
1	17 (36.2)	8 (72.7)	9 (25.0)	
Pn				0.032
0	24 (51.1)	2 (18.2)	22 (61.1)	
1	23 (48.9)	9 (81.8)	14 (38.9)	
Tumor differentiation				0.299
G1	4 (14.8)	1 (20.0)	3 (13.6)	
G2	8 (29.6)	0 (0.0)	8 (36.4)	
G3	15 (55.6)	4 (80.0)	11 (50.0)	
Peritoneal carcinomatosis				<0.001
1	14 (29.8)	10 (90.9)	4 (11.1)	
0	33 (70.)	1 (9.1)	32 (88.9)	

**Figure 1:**
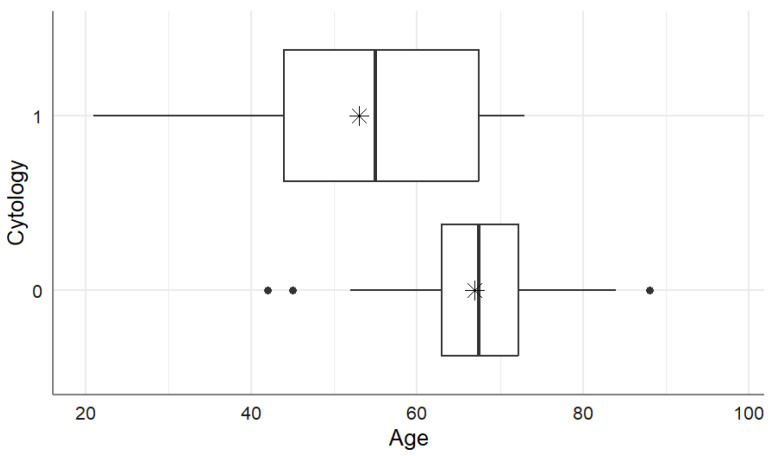
Age distribution according to cytology results. “1” indicates a positive cytology result; “0” indicates a negative cytology result. The mean age values for each group are represented by stars.

A statistical relationship can be observed for the tumor staging, including T (*p* = 0.005), N (*p* = 0.044), M (*p* < 0.001), L (*p* = 0.032), V (*p* = 0.009), and Pn (*p* = 0.032). A relationship was also observed for the level of Ca19-9 collected from abdominal washing fluid (*p* < 0.001) and for pCEA (*p* < 0.001), as well as in patients with visible intraperitoneal metastases (*p* < 0.001). Moreover, the mean age of patients in the group in which cytology did not show cancer cells was higher than the mean age of patients in the group with positive cytology results. This means that the mean age of patients with a negative cytology result was statistically significantly higher compared with the mean age of patients with a positive cytology result (*p* = 0.012) (Figure 1).

### 3.2. Factors Affecting the Development of Peritoneal Carcinomatosis

In the study group, 14 patients (29.700%) were diagnosed with peritoneal carcinomatosis, of which 10 patients had a positive cytology results. A statistically significant relationship was observed with cytology (*p* < 0.001), tumor stage T (*p* < 0.001), N (*p* = 0.012), L (*p* = 0.003), V (*p* = 0.003), Pn (*p* = 0.020), prior neoadjuvant chemotherapy (*p* = 0.022), and blood hemoglobin level (*p* = 0.02). Interestingly, sCEA and sCa19-9 did not show a significant relationship, which, however, can be observed for pCEA (*p* < 0.001) and pCa19-9 (*p* < 0.001) (Table 3).

### 3.3. Serum and Peritoneal Tumor Markers

**Figure 2:**
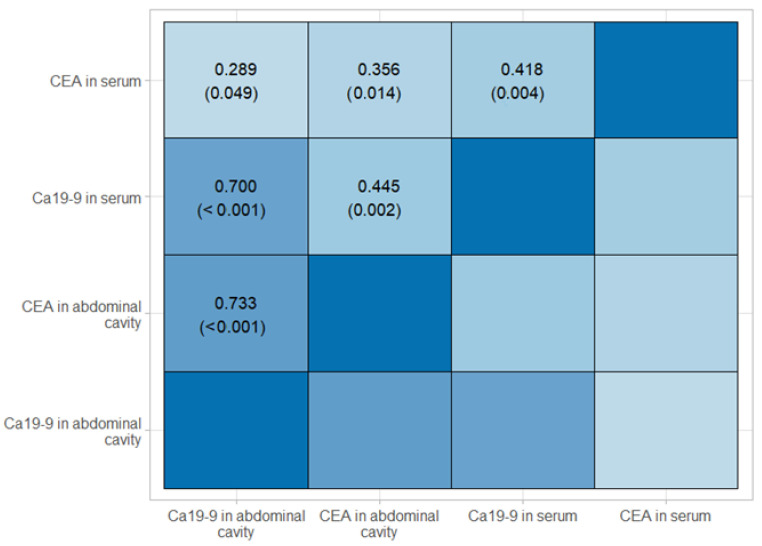
Correlation between sCEA and pCEA levels (Spearman’s rank correlation).

Normal levels for the tumor markers in blood serum were adopted per the laboratory guidelines: sCEA (≤3 ng/mL) and sCa19-9 (≤37 µ/mL). In total, 15 (31.9%) patients had elevated sCEA levels, and 12 (25.5%) had elevated Ca19-9 levels, 9 of whom had elevated levels of both sCEA and sCa19-9. There was no significant relationship between the serum tumor marker levels (sCEA and sCa19-9) and the demographic data, previous neoadjuvant chemotherapy, and tumor features, nor with positive peritoneal cytology. There was also no relationship with morphology or kidney function (based on creatinine or GFR levels) (*p* > 0.05 for all). However, a significant correlation was found between sCEA and pCEA levels (*p* < 0.001 in Fisher’s exact test; Spearman’s rank correlation of 0.418 at *p* = 0.004) (Figure 2).

**Figure 3:**
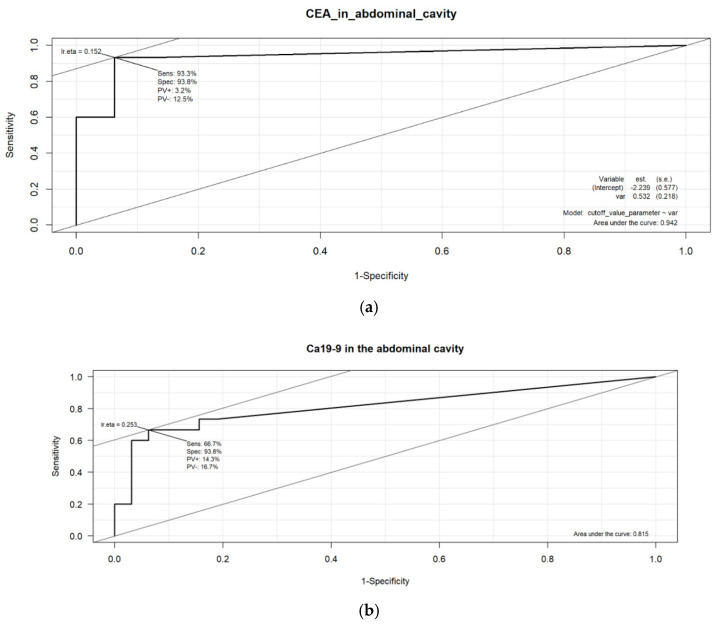
The optimal cut-off point selection for sensitivity relative to specificity was performed using the ROC curve and the AUC value. (**a**) CEA and (**b**) Ca19-9 in the abdominal cavity.

A receiver operating characteristics (ROC) analysis was performed to determine the cut-off values for the peritoneal markers (pCEA and pCa19-9)(Figure 3). Considering sensitivities and specificities, the values were established based on the diagnosis of peritoneal dissemination made at surgery or a positive cytology result. The analysis revealed that the optimal cut-off values for pCEA and pCa19-9 were 0.98 ng/mL and 6.4 µ/mL, respectively. The corresponding sensitivities and specificities under these cut-off values are presented in Table 4.

Based on the results in Table, it can be concluded that pCEA detects tumor cells with high probability. This marker has high sensitivity and specificity (93.3% and 93.8%, respectively). pCa19-9 shows a worse performance. In the case of a correct cancer diagnosis, it obtains a value of 66.7% at the cut-off value of 6.4; however, it recognizes the absence of cancer cells very well—at the same level (93.8%) as pCEA. Grouping based on these cut-off values revealed 16 (34.0%) patients with high pCEA levels and 12 (25.5%) with high pCa19-9 levels.

Significant correlations were observed between pCEA and pCA19-9 (*p* < 0.001 in Fisher’s exact test) and between sCEA and pCEA (*p* = 0.019) and pCa19-9 and sCa19-9 (*p* < 0.001). Significant relationships were also found between the exact numerical values of these markers. A monotonic relationship was demonstrated for each of the marker pairs. The most strongly correlated were pCa19-9 with sCa19-9 (*p* < 0.001) and pCa19-9 with pCEA (*p* < 0.001).

**Figure 4:**
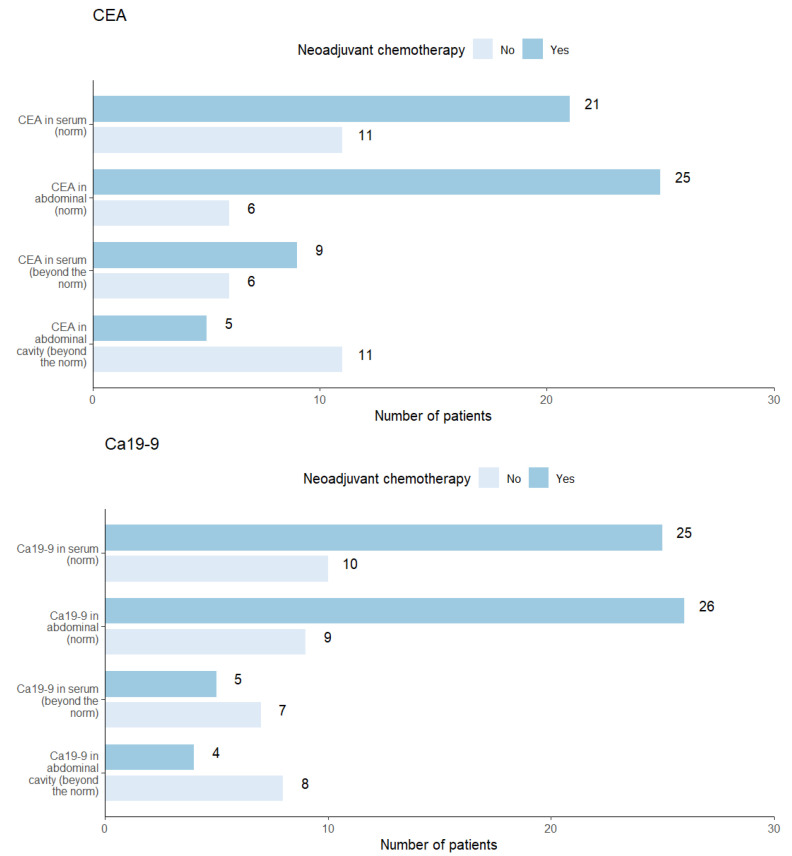
Graphical presentation of the distributions between chemotherapy and the individual markers.

A comparison of the group of patients who underwent neoadjuvant chemotherapy with the group that did not receive it showed no significant differences in the sCEA and sCa19-9 levels (*p* > 0.05 for both). However, significant differences were observed in the statistics for pCEA (*p* = 0.003) and pCa19-9 (*p* = 0.016). This indicates that patients who received neoadjuvant chemotherapy demonstrated statistically significantly lower values for the peritoneal markers compared with the control group (Table 5). The graphical presentation of the distributions between chemotherapy and the individual markers can be observed in Figure 4.

No significant correlation was observed between sCEA and sCa19-9 levels and the demographic data, tumor characteristics, and results of the abdominal fluid cytology. There was also no relationship with blood counts or kidney function (measured via creatinine or GFR levels). For all these results, *p* > 0.05. However, for the markers in peritoneal washing fluid, a statistical relationship was found between pCEA and the tumor features T (*p* = 0.001), N (*p* = 0.006), L (*p* = 0.024), V (*p* = 0.003), and Pn (*p* = 0.024) and the WBC (*p* = 0.02), and the HGB (*p* = 0.027) blood count; moreover, for Ca19-9, a relationship was noted between tumor features T (*p* = 0.01) and V (*p* = 0.02), as well as HCT (*p* = 0.003) and PLT (*p* = 0.039). A statistically significant relationship was also found between the pCa19-9 feature and the patient’s age (*p* = 0.007). However, no association was observed between pCEA or pCa19-9 and the histological cancer type.

## 4. Discussion

Unfortunately, a high percentage of patients treated surgically with curative intent later struggle with cancer recurrence, which often ends in death [19].

Given the aggressiveness of gastric cancer, early diagnosis is a crucial step toward achieving long-term survival. This has been confirmed by the improved survival rates in Asian countries, where population screening has been introduced [20]. Staging laparoscopy and peritoneal cytology are recommended tools for detecting latent peritoneal dissemination, especially in patients at an early disease stage [5,21].

Some centers supplement laparoscopy with peritoneal washing and cytological examination in the absence of ascites or macroscopic signs of neoplastic dissemination. This is because patients with positive cytology results have a worse prognosis and a higher risk of cancer recurrence [10]. The employment of laparoscopic ultrasonography to increase the staging accuracy has also been proposed, but there are few studies on this procedure [22].

There have also been attempts to use indocyanine green in infrared light, which has shown promising results. However, very limited clinical data are available, and all of them concern retrospective studies in which this technique was used during cytoreductive surgery in patients with known peritoneal metastases [23].

It is difficult to accurately find small cancer metastases in the peritoneal cavity in patients with gastric cancer. Conventional cytology remains crucial for diagnosis and is used to detect free cancer cells. However, classic cytology techniques may face difficulties. The limited number of cells exfoliated into the peritoneal cavity (until the advanced stage of the disease) and their similarity to reactive mesothelial or inflammatory cells in exudative fluids reduce the ability to detect cancer cells using cytology. Moreover, pathologists make diagnoses based solely on cell morphology. Therefore, without considering histological analyses, a pathological diagnosis may be delayed or inaccurate [24]. Invasive procedures improve accuracy but are limited by their invasiveness and diagnostic efficiency.

Some studies have tried to demonstrate a relationship between tumor markers in serum and peritoneal fluid and the cancer stage. Attempts were made to demonstrate their usefulness in determining the cancer stage and the risk of recurrence [1,2,3,4]. For gastric cancer, CEA and Ca19-9 are the most frequently studied markers [4]. The levels of these two tumor markers in serum and the peritoneum were used in this study.

These markers can serve as an auxiliary tool in the diagnosis and clinical assessment of a patient [1,2,3]. These markers, both individually and in combination, have been shown to increase the sensitivity of detection of lymphatic and peritoneal metastases in gastric cancer [1,25].

Hasbahceci’s study [1] showed that serum CEA levels are significantly associated with an unfavorable prognosis in patients with gastric cancer. Serum Ca19-9 levels and high peritoneal CEA concentrations are significant indicators of a positive cytology result and the development of peritoneal carcinomatosis, respectively. Only sCa19-9 was shown to be significantly associated with the tumor diameter and the TNM grade. However, no such association was detected for sCEA. This study did not demonstrate such relationships for either sCEA or sCA19-9.

Yamamoto’s results [2] suggest that the CEA level in peritoneal lavage fluid is an indicator of peritoneal dissemination. Another study [3] showed that pCEA, pCA72-4, and pCA72-4 levels are independent predictors of peritoneal dissemination. In our study, both pCEA and pCa19-9 were also statistically associated with intraperitoneal dissemination, as opposed to sCEA and sCa19-9.

A systematic review found that preoperative serum tumor markers (CEA, CA19-9, and CA72-4) are significantly associated with the tumor stage and patient survival [4]. However, this was not confirmed in our study. The same study did not prove a relationship between the values of these markers and peritoneal recurrence.

Another meta-analysis showed that a high sCEA concentration, as an independent prognostic factor for gastric cancer, doubles the risk of mortality [26].

Some studies have tried to establish a relationship between the results of tumor markers collected from blood serum and those taken from fluid from the peritoneal cavity with their roles in different environments. In Fang Liu et al.’s study [27] with established cut-off values for tumor markers, tumor markers in the peritoneal fluid showed better diagnostic efficacy than those in the blood. The use of a combination of tumor markers and cytology increased the diagnostic result of cytology by 37%. In cases of a negative cytology result collected for ascites due to tumor metastasis, tumor markers helped in distinguishing malignant ascites from benign ascites, and the combination of tumor markers in the peritoneal fluid achieved a sensitivity of 86% and a specificity of 97%.

In cases where peritoneal carcinomatosis cannot be diagnosed with conventional imaging techniques, pCEA has been found to have higher sensitivity than cytology in detecting peritoneal carcinomatosis. In Yamamoto’s study [2], the sensitivity and specificity of pCEA were 75.8% and 90.8%, respectively.

Hasbahceci et al. [1] showed that only serum CEA (sCEA) levels were significantly associated with disease-free survival and overall survival (*p* = 0.002 and *p* = 0.001, respectively). Although Cox regression analysis did not show a significant association between pCEA and disease-free survival and overall survival, the Kaplan–Meier analysis revealed poorer survival in patients with high pCEA levels.

Our study demonstrated that CEA and Ca19-9 in abdominal fluid are valuable in staging gastric cancer. pCEA showed both high sensitivity (93.3%) and specificity (93.8%). pCa19-9 performed slightly worse, with a sensitivity of 66.7% but a specificity of 93.8%, similar to that of pCEA. Both tumor cut-off values were determined based on the ROC curve (Table 4). These relationships were not observed for the same serum markers.

Tuzun et al. [28] found that tumor marker levels were highly correlated in serum and peritoneal fluid in a group of malignant tumors, and the detection of tumor markers in peritoneal fluid alone had no advantage over serum analysis. Our results showed that only markers from the peritoneal fluid were associated with the cancer stage. The studies by Tuzun [28] and Chen [29] showed that the average levels of CEA were much higher in the fluid from the peritoneal cavity than in the serum in their group of malignant tumors; this indicates that collecting tumor markers from the abdominal cavity may be justified, as it may be associated with better diagnostic efficiency. Therefore, the measurement of tumor markers in the fluid from the peritoneal cavity should be recommended in clinical practice when malignant ascites is suspected.

Fang Liu [27] showed that tumor markers collected from the abdominal cavity have a high diagnostic efficiency in differentiating ascites. The diagnostic efficacy of ascitic CEA or Ca19-9 individually was comparable to cytology, while the combination of CEA, Ca19-9, and CA15-3 provided better sensitivity (85%) and specificity (97%) compared with CEA, Ca19-9, and CA15-3 alone. The addition of tumor markers to the cytological analysis resulted in a 37% increase in the diagnostic rate, with little impact on the specificity. In our study, we did not show a statistical relationship between sCEA and sCa19-9 levels and a positive cytology result or the presence of intraperitoneal dissemination; however, this did not apply to pCEA and pCa19-9.

Several factors may explain the correlation between a younger age and positive peritoneal cytology in gastric cancer. Younger patients are more likely to have genetic predispositions or familial syndromes associated with an aggressive disease, leading to a higher risk of peritoneal spread. Additionally, gastric cancer in this age group often presents with nonspecific symptoms, resulting in delayed diagnosis and advanced disease at the time of detection, which increases the risk of metastasis. Tumors in younger patients may also exhibit higher proliferation rates, making them more aggressive and prone to early metastasis. Furthermore, differences in immune responses between younger and older patients could facilitate quicker peritoneal seeding by cancer cells. Collectively, these factors contribute to the observed correlation

The limitations of our project mainly include the limited study group size and the use of only two tumor markers. The lack of cut-off values for the levels of peritoneal tumor markers and the calculation of these values using the study group are other drawbacks of this study. Although the use of a prospective database was an important aspect of this study’s accuracy, larger study groups are needed to achieve more meaningful results.

## 5. Conclusions

In conclusion, this study showed that using serum and peritoneal tumor markers for the diagnosis and prognosis of gastric cancer remains controversial. Nevertheless, the examination of tumor markers from abdominal lavage fluid (pCEA and pCa19-9) shows a significant association with the cancer stage. In addition, the level of the above markers is an important predictor of positive peritoneal washing cytology. The detection of tumor markers can complement cytology in the diagnosis of gastric cancer staging. Therefore, the potential impact of CEA and Ca19-9 in serum and peritoneal fluid (especially on the diagnosis of cancer progression) should be evaluated in future large-scale prospective studies.

## Figures and Tables

**Table 1:** The demographic and clinical data. Abbreviations: SD—standard deviation; med—median; iqr—interquartile range.

Variables	Mean (SD)	Med (iqr 25–75%)
Age (years)	63.68 (13.34)	67.00 (57.50–71.00)
CEA in serum (ng/mL)	8.66 (30.03)	2.10 (1.30–4.35)
CEA in abdominal cavity (ng/mL)	24.20 (70.14)	0.00 (0.00–4.15)
Ca19-9 in serum (U/mL)	626.52 (2299.21)	8.20 (2.95–40.95)
Ca19-9 in abdominal cavity (U/mL)	575.32 (2444.11)	0.00 (0.00–6.50)
WBC (10^3^/μL)	7.36 (2.33)	7.15 (6.11–8.22)
HGB (g/dL)	11.66 (2.01)	11.50 (10.50–13.00)
HCT (%)	36.19 (5.57)	36.40 (33.60–39.95)
PLT (10^3^/μg)	241.75 (86.36)	235.20 (192.65–265.65)
Creatine (mg/dL)	0.86 (0.36)	0.79 (0.66–0.99)
GFR (mL/min/1.73m^2^)	94.76 (28.67)	92.80 (78.43–111.30)

**Table 3:** Number of patients with detected indicators: carcinomatosis, positive cytology, elevated pCa19.9, and pCEA.

	Carcinomatosis	Positive Cytology	Positive pCa19.9	Positive pCEA
Carcinomatosis	14	10	9	13
Positive Cytology	10	11	9	10
Positive pCa19.9	9	9	14	14
Positive pCEA	13	10	14	18

**Table 4:** Sensitivities and specificities of CEA and Ca19-9 in abdominal cavity washings.

Marker	AUC	CUT-OFF	Sensitivity	Specificity
CEA in abdominal cavity	0.942	0.98	0.933	0.938
Ca19-9 in abdominal cavity	0.815	6.40	0.667	0.938

**Table 5:** The statistical relationship between chemotherapy and individual markers. A value of “1” for chemotherapy indicates that the patient underwent neoadjuvant chemotherapy, while “0” indicates that the patient did not.

Variables, n (%)	Chemotherapy		*p*-Value
	1	0	
Number of subjects	30 (63.8)	17 (36.2)	
CEA in serum			0.961
Norm	21 (70.0)	11 (64.7)	
Beyond the norm	9 (30.0)	6 (35.3)	
CEA in abdominal cavity			0.003
Norm	25 (83.3)	6 (35.3)	
Beyond the norm	5 (16.7)	11 (64.7	
Ca19-9 in serum			0.087
Norm	25 (83.3)	10 (58.8)	
Beyond the norm	5 (16.7)	7 (41.2)	
Ca19-9 in abdominal cavity			0.016
Norm	26 (86.7)	9 (52.9)	
Beyond the norm	4 (13.3)	8 (47.1)	

## Data Availability

The data presented in this study are available on request from the corresponding author due to legal (internal hospital data, where the study took place) and ethical reasons.

## Supplementary Materials

The following supporting information can be downloaded at: https://www.wcrf.org/cancer-trends/stomach-cancer-statistics/ (accessed on 6 November 2024), https://www.iarc.who.int/news-events/the-current-and-future-incidence-and-mortality-of-gastric-cancer-in-185-countries-2020-40-a-population-based-modelling-study/ (accessed on 6 November 2024).
